# P08 Preventing and managing urinary tract infections: using thematic analysis to explore interventions and strategies implemented by NHS commissioning organizations in English primary care, 2017–22

**DOI:** 10.1093/jacamr/dlad077.012

**Published:** 2023-08-02

**Authors:** Eirwen Sides, Donna Lecky, Esther Taborn, Luke O’Neill, Emily Cooper

**Affiliations:** UK Health Security Agency, UK; UK Health Security Agency, UK; NHS England & Improvement, UK; UK Health Security Agency, UK; UK Health Security Agency, UK

## Abstract

**Objectives:**

To identify and explore strategies, interventions and activities used by English Clinical Commissioning Groups (CCGs) to improve prevention and management of urinary tract infections (UTIs) from 2017–22.

**Methods:**

Online questionnaires were sent to primary care chief nurses and medicines optimization leads via regional infection prevention control (IPC) and antimicrobial stewardship (AMS) leads August–September 2022. Qualitative data were mapped to the Theoretical Domains Framework.

**Results:**

Response rate was 14.1% (56/397 participants approached), with representation from 29.2% (31/106) English CCGs. Respondents provided 201 examples of UTI interventions. Themes identified included: (i) populations and settings; (ii) aspects of UTI targeted; (iii) types and functions of interventions and activities; (iv) resources, tools and policies; and (v) implementation strategies. Education and training were the most used type of intervention, while changing the environment to facilitate ideal behaviours was the least used intervention type. Most interventions targeted general practice staff and patients accessing primary care, followed by care home staff and residents and their families. The most used success measures included reduction in antibiotic prescribing (54.5%, 97/178 intervention examples); positive feedback from stakeholders (42.1%, 75/178); and increased adherence to national diagnostic guidelines (32.6%, 58/178). However, 48.8% (20/41) stated their team’s UTI activities had not been evaluated, indicating that success was determined using informal data or perceived outcomes. Barriers to implementation of UTI interventions included: lack of resources and time; low staff engagement, inaccurate data due to lack of clinical coding; inconsistent systems and processes; and lack of dedicated roles. Facilitators to implementation included: availability of tools; financial incentives; multidisciplinary collaboration; sharing of information; staff motivation; embedding resources into systems; digitalization; and dedicated roles.

**Conclusions:**

Multidisciplinary teams and cross-sector collaboration are key facilitators for implementation of UTI interventions. Although some teams are already using robust success measures, there is a need to improve how interventions are evaluated to inform resource allocation and planning. Education and training interventions should address or leverage the barriers and facilitators identified, including clinical coding skills, embedding resources and tools into existing systems and auditing adherence to diagnostic and antibiotic prescribing guidance. There may be missed opportunities for intervention types that are less widely used e.g. changing environmental factors.

